# Development, Implementation, and Evaluation of a Faculty Scholarship Program for Emergency Medicine Clinician Educators

**DOI:** 10.1002/aet2.70238

**Published:** 2026-08-03

**Authors:** Amina Belghit, William B. Stubblefield, Erik P. Hess, Jeremy S. Boyd, Deja M. Curry, Jim Olechna, Alan B. Storrow

**Affiliations:** ^1^ Department of Emergency Medicine Vanderbilt University Medical Center Nashville Tennessee USA

**Keywords:** curriculum improvement, faculty development, implementation science, PRISM framework, protected time

## Abstract

**Background:**

Scholarship is essential for career progression within academic settings. Unfortunately, time pressures, uncertain processes, and unclear or absent resources remain barriers. We describe the development, implementation, and effectiveness of an internally funded scholarship program for EM faculty clinician educators led by the effort from 2 faculty physician scientists and a full‐time Health Science Research Analyst.

**Methods:**

The program was developed through a needs assessment survey, discussions with faculty development leadership, and components of the PRISM implementation framework. Effectiveness was assessed using an interrupted time series (1 year pre‐ and 1 year post‐implementation). Scholarship was defined a priori as manuscripts, abstract acceptances, regional/national presentations, book chapters, and online publications. The target audience was faculty, residents, fellows, attendings, and advanced practice providers.

**Results:**

Our needs assessment survey suggested deficiencies in mentorship, formal curriculum, academic writing, and navigating journal submissions. A conceptual framework for scholarship was developed with 4 domains: education, clinical trials and studies, administrative, and training. The program was implemented July 1, 2024, and adopted by 17% (*n* = 17) of the audience. Acceptability ratings were high, with over 88% of respondents selecting 4/5 or 5/5 (1–5 ordinal scale). Overall scholarship increased from pre‐implementation (146 manuscripts, 69 abstracts, 54 presentations, 27 book chapters, 34 online publications; total 330) to post (195 manuscripts, 90 abstracts, 78 presentations, 28 book chapters, 57 online publications; total 448), representing a 35.8% growth. The most notable increases occurred in manuscripts, followed by abstracts and presentations. For adoptees, output increased from a mean of 0.4 to 0.58 per participant per year.

**Conclusion:**

A faculty scholarship program for EM clinician educators was successfully executed within an implementation science framework. Our program was well received by end users and displayed an increase in scholarly output. Future evaluations will focus on program sustainability, growth, and impact on promotion.

## 1 Introduction

Faculty scholarly output is a widely recognized metric of academic engagement and professional advancement [1]. Unlike formal programs supporting tenure‐track physician scientists, many departments of emergency medicine (EM) lack formalized programs to support non‐tenure track clinician educators in the development and dissemination of scholarly work [1, 2]. Scholarly activities foster a culture of scientific inquiry, enhance clinical knowledge in relation to patient care, and ensure healthcare professionals remain at the forefront of medical advancements [3, 4]. Enhancing faculty scholarship is crucial to help align individual performance and behavior with broader institutional goals [3]. Evidence suggests structured faculty scholarship programs significantly improve research productivity and educational quality across academic departments [1]. Furthermore, national needs assessments highlight persistent gaps in mentorship and institutional support, underscoring the urgency for targeted interventions [3].

Despite the recognition of evolving changes in scholarly pursuits, there is considerable variability in how institutions incorporate comprehensive scholarship initiatives [4]. The lack of infrastructure to support non‐tenure track clinician educators is often a barrier to the development and dissemination of scholarly work in an academic department [3, 4]. Recognizing these potential gaps, targeted strategies must not only address barriers within scholarly support but also create sustainable pathways for academic growth among clinician educators. Faculty scholarship should strive to align individual performance and behavior with an institution's broader goals and outcomes to effectively assess the organization's overall impact [5]. In one study evaluating healthcare professionals' perceptions of faculty scholarship, similar views on the importance of such programs were widely recognized, despite low participation across institutions [6]. Lack of participation in scholarly activities can be attributed to faculty attitudes, insufficient institutional support, and a scarcity of research focused on improving educational practices [7]. We aimed to strengthen the scholarly infrastructure within the department of EM at Vanderbilt Health by identifying clinician educators' scholarship needs, implementing a sustainable support model, and evaluating our primary outcome of scholarly output.

## 2 Methods

### 2.1 Context

Our scholarship program was implemented at a large academic emergency department (ED) at Vanderbilt Health in Nashville, TN. Participants of our program included faculty, residents, fellows, attendings, and advanced practice providers (APPs). The program engaged residents who participated under the mentorship of a faculty member with formal training and experience as a clinician educator. Academic medical centers often have substantial differences in how non‐tenure faculty pathways are labeled and operationalized, despite broadly similar expectations regarding clinical excellence, teaching, and scholarly contribution. Although the term “clinician educator” is commonly used, comparable promotion tracks are defined differently depending on institutional norms and governance structure. For instance, The University of Michigan employs Clinical Track faculty designations to recognize faculty whose primary responsibilities center on clinical care and teaching with expectations for scholarly dissemination that do not hinge on external grant funding [8]. Similarly, Yale School of Medicine coins the term Clinician Educator Scholar Track for faculty who play an integral role in the department's clinical and teaching programs, while also participating in the research endeavors of the institute [9].

Vanderbilt Health has three academic pathways for physicians: tenure track physician scientist, non‐tenure track clinician educator, and non‐tenure clinical practice track. For the purposes of this initiative, we use the term “clinician educator” as an inclusive descriptor for faculty and clinical professionals on non‐tenure academic pathways for whom scholarship is expected but not primarily grant driven. This framing allowed our program to be generalizable and to professional roles and promotion tracks within other institutions.

### 2.2 Comprehensive Needs Assessment

#### 2.2.1 PRISM

Implementation science utilizes theories, models, or frameworks to: (a) guide decisions, hypothesis generation, measure selection, and analyses; (b) enhance generalizability; and (c) improve outcomes or at least understanding [10, 11]. We utilized components of the Practical, Robust Implementation and Sustainability Model (PRISM) to address contextual factors within our implementation strategies (program, practice, policy) and to guide our program development. Our implementation outcomes were reach, adoption, and acceptability. Using components of an implementation science framework ensured our program was evidence‐based, adaptable to faculty needs, successfully implemented within our existing departmental context given available resources, time, and integrated into institutional priorities, creating a foundation for long‐term sustainability.

### 2.3 Components of Our Needs Assessment

The science of dissemination and implementation is a growing field which commonly involves change at multiple levels at both outer (i.e., system) and inner (i.e., organization) contexts of service systems and organizations [12]. One important consideration for our needs assessment was how leadership influences implementation across multiple organizational levels within academic health care settings, as variability in leadership roles can directly affect mentorship availability and access to academic resources [12, 13, 14]. Balancing teaching responsibilities with research and professional development is a significant challenge when there is no allocated protected time for faculty to engage in scholarly pursuits. Although faculty scholarship programs are widely available across academic institutes, our program uniquely developed a local needs assessment to identify barriers to scholarly productivity among clinician educators and early‐career faculty, including limited mentorship access, insufficient protected time and lack of structured accountability.

The development process involved a multi‐step approach including content development initially generated based on a review of relevant literature, existing assessment frameworks, and internal departmental priorities. Stakeholder engagement was refined through feedback from departmental leadership to ensure content validity and relevance to the development of our program. With support from departmental leadership, the scholarship program was developed using an implementation science framework to guide program design, integration, and sustainability. Our key focus was to (1) determine the level of knowledge needed in each discipline; (2) identify the necessary skills required; and (3) assess the individual needs of clinical educators. This structured approach helped identify discrepancies between the current state of our department and our desired outcomes. Data were collected through physician needs assessment surveys, discussions with faculty development leaders, and analysis of contextual factors, including prior initiatives and available resources. A needs assessment survey was distributed to all faculty members of the department using a structured dissemination strategy (REDCap survey) to maximize response rates and representation. In addition to demographics we collected text responses for the top 2 needs for each faculty, giving examples within categories such as education, administration, advocacy, training, regulatory, academic writing, methodology, and analysis. We aimed to focus on defining faculty scholarship, developing a conceptual framework, analyzing our primary outcome of scholarly output, and assessing individual faculty needs. Our findings from our needs assessment shaped both the structure and content of our program, making it responsive to the real‐world needs of clinician educators rather than based on a generalized faculty development framework. A reproducible model is provided as Table 1.

**TABLE 1:** This reproducible model provides a framework that may be adapted by other institutes to strengthen scholarly productivity and engagement.

Strategy	Needs assessment barrier(s) addressed
Dedicated effort from 2 faculty physician scientists and a Health Services Research Analyst	Insufficient mentorship; limited research expertise among clinician educators; lack of guidance for project development, study design, data analysis, manuscript preparation, and scholarly dissemination
Faculty development web link	Poor awareness of available resources; fragmented access to scholarship support; need for centralized tools, templates, and educational resources
Yearly workshop for new faculty and fellows	Deficiencies in formal curriculum, academic writing, peer‐reviewed publishing, conference presentations, and research skill development
REDCap intake form	Need for individualized assessment of scholarly goals, project scope, mentorship requirements, regulatory support, and research assistance needs
Individual faculty meeting with implementors	Limited knowledge of available departmental resources; need to increase program awareness, engagement, and adoption among faculty and clinicians
Faculty scholarship conceptual model	Unclear scholarship processes; difficulty navigating project development, institutional resources, mentorship pathways, and dissemination activities

### 2.4 Measures

Our primary outcome measure was scholarly output defined as all manuscripts, abstract acceptances, regional/national presentations, book chapter publications, and online scholarly publications. This metric also serves to quantify productivity in ways which are directly aligned with criteria used for academic promotion. Scholarly output was assessed using an interrupted time series: 1 year pre‐implementation (July 1, 2023–June 30, 2024) and 1 year post‐implementation (July 1, 2024–June 30, 2025). A multifaceted approach was used to assess scholarship data through updated faculty CVs, PubMed searches, and ORCID profiles. Secondary measures included program adoption and acceptability, consistent with the PRISM implementation framework. Adoption was defined as participation in at least one scholarly project supported by the program. Acceptability outcomes were summarized descriptively. Acceptability was assessed via post‐participation survey using a 5‐point Likert scale. Survey items were developed by program leaders to assess participants' perceptions related to mentorship, accessibility of resources, and overall impact of the program on scholarly productivity. The survey was not externally validated, but went through internal pilot testing with faculty development leaders to evaluate item clarity, content relevance, and usability. The survey was distributed to program participants and aimed to assess perceived effectiveness and impact. Six multiple‐choice questions were structured to capture quantitative data on various aspects of engagement in the program, including perceived benefits, areas for improvement, and resources utilized. Two free‐text response questions were designed to allow participants to provide qualitative feedback on suggestions for improvement of the program. The survey was developed using REDCap, a secure web application created at Vanderbilt University for constructing and managing online surveys and databases [6, 15]. Survey data were securely stored on REDCap and remained accessible only to authorized study personnel. The survey received approval from the Vanderbilt University Medical Center Institutional Review Board (IRB) as exempt with a waiver of informed consent.

### 2.5 Data Analysis

Scholarly output data were analyzed descriptively, with proportions to quantify the distribution of responses across various categories and identify prevalent patterns. A single trained reviewer systematically extracted and verified all scholarly outputs to ensure consistent coding. To optimize data reliability, counts were cross‐referenced across three sources: updated faculty CVs, PubMed entries, and ORCID records. When discrepancies occurred (e.g., missing or outdated ORCID profiles), CV and PubMed data were used as authoritative sources. All authorship contributions were counted equally regardless of authorship position.

To evaluate program impact, we calculated the change in total scholarly output between the pre‐ and post‐implementation time frames, as well as category‐specific changes in manuscripts, abstracts, presentations, book chapters, and online publications. For adoptees of our program, mean individual scholarly output was calculated for both time periods to assess whether concentrated support was associated with increased productivity at the individual level. Adoption and acceptability were analyzed within the PRISM/RE‐AIM framework. Adoption was recorded as a binary variable reflecting participation in at least one supported scholarly activity. Acceptability was analyzed descriptively based on Likert‐scale responses from the post‐participation survey.

To further conceptualize the program's impact, we wanted to understand how scholarly productivity changed among the various clinician educator groups following implementation. Specifically, we evaluated scholarly output across pre‐implementation and post‐implementation periods for all faculty, fellows, residents, attendings, and APPs. Physician scientists were intentionally excluded from this evaluation because of their advanced research training, dedicated protected time, and established scholarly infrastructure, which positioned them outside the program's intended target population. Their exclusion allowed for a clearer evaluation of the program's effect on the clinician educator cohort most likely to benefit from structured scholarly support.

## 3 Results

Discussions with various stakeholders in the department of EM revealed several critical barriers to scholarship: (1) an insufficient structured mentorship program; (2) limited opportunities for interdisciplinary collaboration; (3) lack of advanced research skills among clinical educators; and (4) insufficient knowledge of available resources within the department. We addressed these identified gaps by defining the necessary actions to meet the prioritized needs of our target audience. Our needs assessment survey highlighted priorities in quality improvement initiatives, curriculum development, mentorship structure, strategies for national leadership and conference presentations, regulatory support, and focused guidance on peer‐reviewed publishing.

**FIGURE 1:**
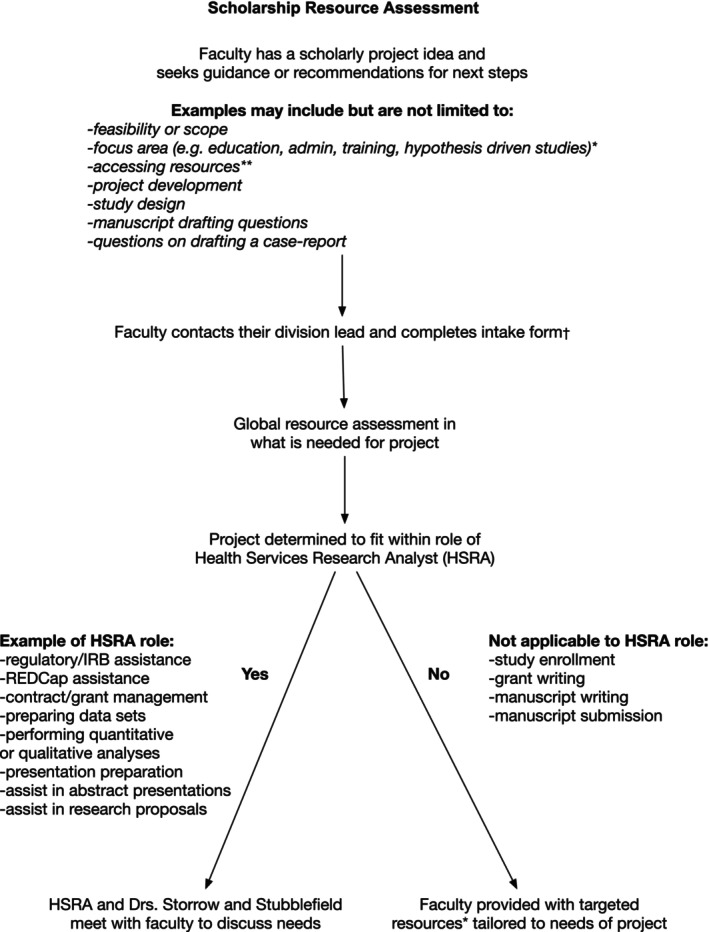
This process model outlines the available support for our faculty, including but not limited to regulatory support, presentation preparation, performing qualitative and quantitative analyses, and assistance in developing research proposals.

Following our needs assessment survey, we developed a strategy to successfully execute our program. First, we dedicated partial effort from 2 faculty physician scientists and a full‐time Health Services Research Analyst. Then, a faculty scholarship web link was created to make resources easily accessible. The program was presented at our monthly faculty meeting, and a yearly workshop for fellows, new faculty, and new attendings was delivered. A faculty scholarship process (Figure 1) was created to outline the key steps for our clinician educators seeking to conduct scholarly pursuits.

At last, a REDCap intake form was created and made accessible on the department website. The intake form included brief questions to understand the scope of work and research support needed by our clinician educators.

### 3.1 Faculty Scholarship Model

Faculty scholarship teaching and learning methods are often limited to workshops and seminars, while more diverse methods, such as simulation‐based learning, peer observation, mentoring, and non‐face‐to‐face online learning, remain underutilized [16]. In our efforts to develop a comprehensive program to support scholarly endeavors, we acknowledged the need to prioritize faculty awareness of available resources. A faculty scholarship model (Table 2) was established to examine the existing support mechanisms available within our department, encompassing areas in education, clinical trials and studies, administration, training programs, and various research committees. The model was designed to diversify faculty teaching and learning methods while strategically aligning all available resources with each individual's specific goals and project needs. For example, one educational resource available within the department is the Vanderbilt Health educator development program [17]. The goal of the educator development program is to enhance the knowledge, attitudes, and skills of all faculty members (physicians, nurses, and scientists), learners (students, residents, and fellows), and others (administrators, department leaders) who teach in the medical graduate or nursing school environment. *Society of Academic Emergency Medicine* (SAEM) committees are available for faculty participation, including several focused on consultation services, education, faculty scholarship, fellowship approval, finance, and grants [18]. In organizing available support within a coherent structure, the faculty scholarship model functioned not only as a roadmap but also as a mechanism to enhance departmental culture around scholarship. A tailored, multimodal learning approach ensured faculty scholarship activities were directly linked to measurable scholarly outputs. This strategy was essential for building sustainable academic momentum among clinician educators, particularly those with heavy clinical workloads and limited prior exposure to structured scholarly support programs.

**TABLE 2:** The structured educational, research, administrative, and training available within the department to support clinician educators in developing, executing, and disseminating scholarly work.

Emergency medicine faculty scholarship model			
	Foci	Products	Resources
Education	Assessment Competency development Curriculum development Evaluation Faculty development Instructional strategies Leadership Learning environment Simulation Wellness CME	Protocols Lectures Curriculum Abstracts Case reports/series Manuscripts Quality improvement Educational hypothesis driven research	ACEP/CORD Teaching Fellowship AAMC Medical Education Research Certificate (MERC) Southern Group on Education Affairs (SGEA) Faculty Incubator (Academic Life in EM) VUMC Educator Development Program SAEM EMF AMA SAEM Master Educator Fellowship Advanced Research Methodology Evaluation and Design (ARMED) MedEd SAEM
Clinical trials and studies, hypothesis driven	Cardiovascular Critical care/ID Geriatrics Operations Ultrasound Implementation Qualitative	Grants Abstracts Manuscripts Modules	VICTR SRC (voucher, expedited, full) Other VICTR, studios, biostats clinic MPH/MSCI Federal
Administrative	Clinical operations Quality improvement Process improvement Patient safety	Protocols AgileMD Manuscripts QI Leadership training	ACEP and SAEM Leadership Academies ACEP Emergency Department Directors Academy (EDDA) Tennessee Medical Association Physician Leadership Lab ABEM Healthcare Administration Leadership and Management certification VUMC Mid‐Career Skills Program VUMC Academic Leadership Program AACEM Chair Development Program SAEM education courses Owen School of Business
Training	Clinical research Procedures Global health EMS Simulation VA quality scholars Ultrasound	Mentored training grant (K23, K08) VICTR VFRS Other VICTR resources Curriculum	Advanced Research Methodology Evaluation and Design (ARMED) SAEM Federal—T32, K12, DoD Owen School of Business

*Note:* The Vanderbilt Institute for Clinical and Translational Research (VICTR) is one key resource bridging all clinical and translational research within VUMC. VICTR provides the necessary tools and support systems to enhance the quality of research, training, and grant writing. Additionally, VICTR offers vouchers for biostatistical support and other core services such as qualitative analysis and laboratory tests. Version 6—Alan Storrow.

**FIGURE 2:**
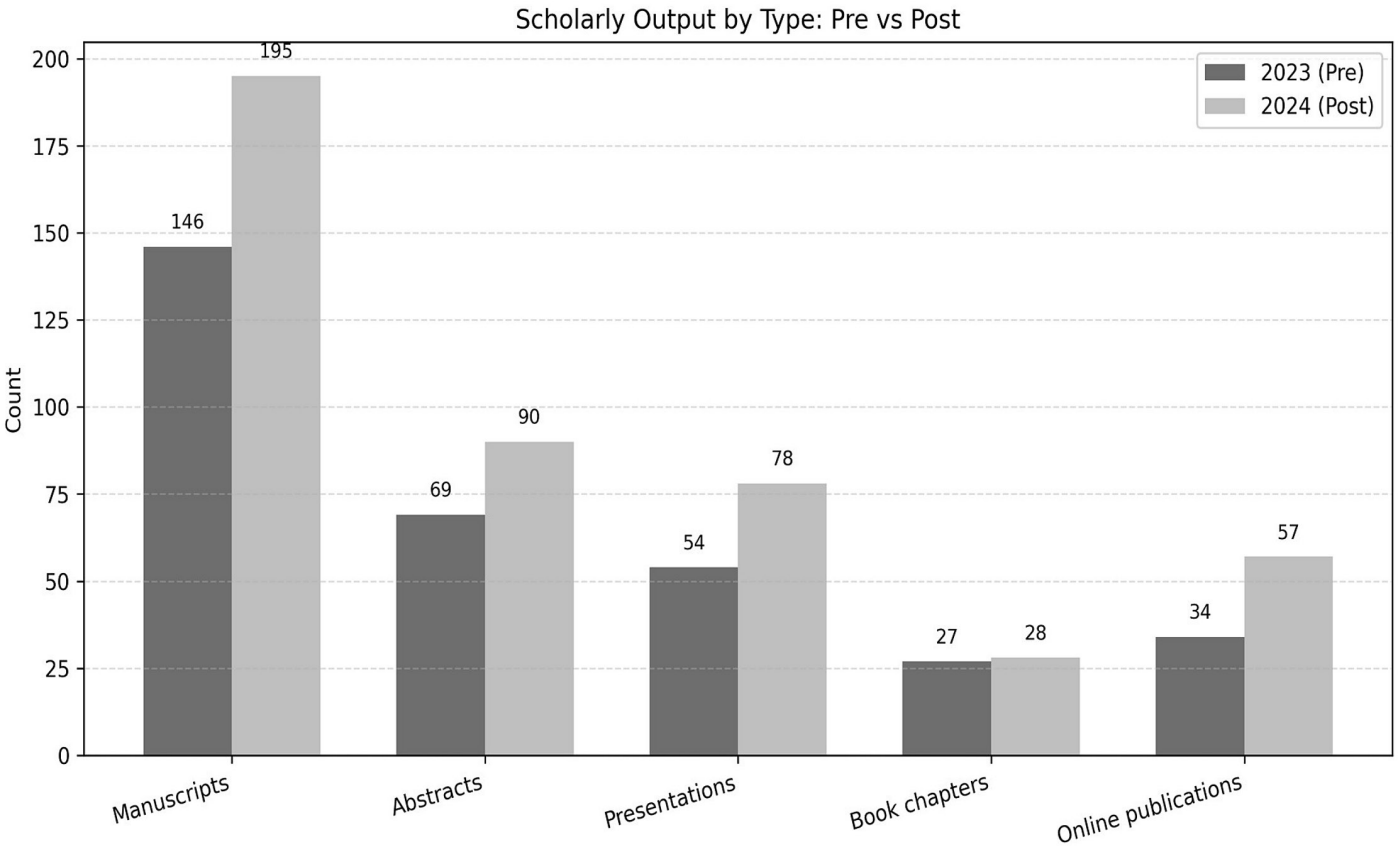
Manuscripts demonstrated the largest increase, rising from *N* = 146 to *N* = 195. Abstract acceptances increased from *N* = 69 to *N* = 90, while regional and national presentations increased from *N* = 54 to *N* = 78. Book chapter publications increased minimally from *N* = 27 to *N* = 28. Online scholarly publications increased from *N* = 34 to *N* = 57 during the post‐implementation period.

Following program implementation July 1, 2024, 17% of the eligible audience, including nine faculty, three fellows, one resident, three attendings, and one APPs (*N* = 17 of 100 in target audience) participated in the faculty scholarship program in Year 1. Participants included faculty, residents who participated under the mentorship of a clinician educator faculty member, fellows, attendings, and APPs. Program acceptability was high, with over 88% of survey respondents rating the program a 4 or 5 on a 5‐point Likert scale. Total department scholarly output increased from 330 in the pre‐implementation period (2023) to 448 in the post‐implementation period (2024). Increases were observed across all predefined categories of scholarship (Figure 2).

Among individuals who adopted the program, the mean scholarly output increased from 0.4 in the pre‐implementation period to 0.58 per clinician educator (1 output per 30 months to 1 output per 20 months) in the post‐implementation period. Across the post‐implementation period, manuscripts and abstracts accounted for most academic outputs, while online publications constituted the largest growth in total volume.

**FIGURE 3:**
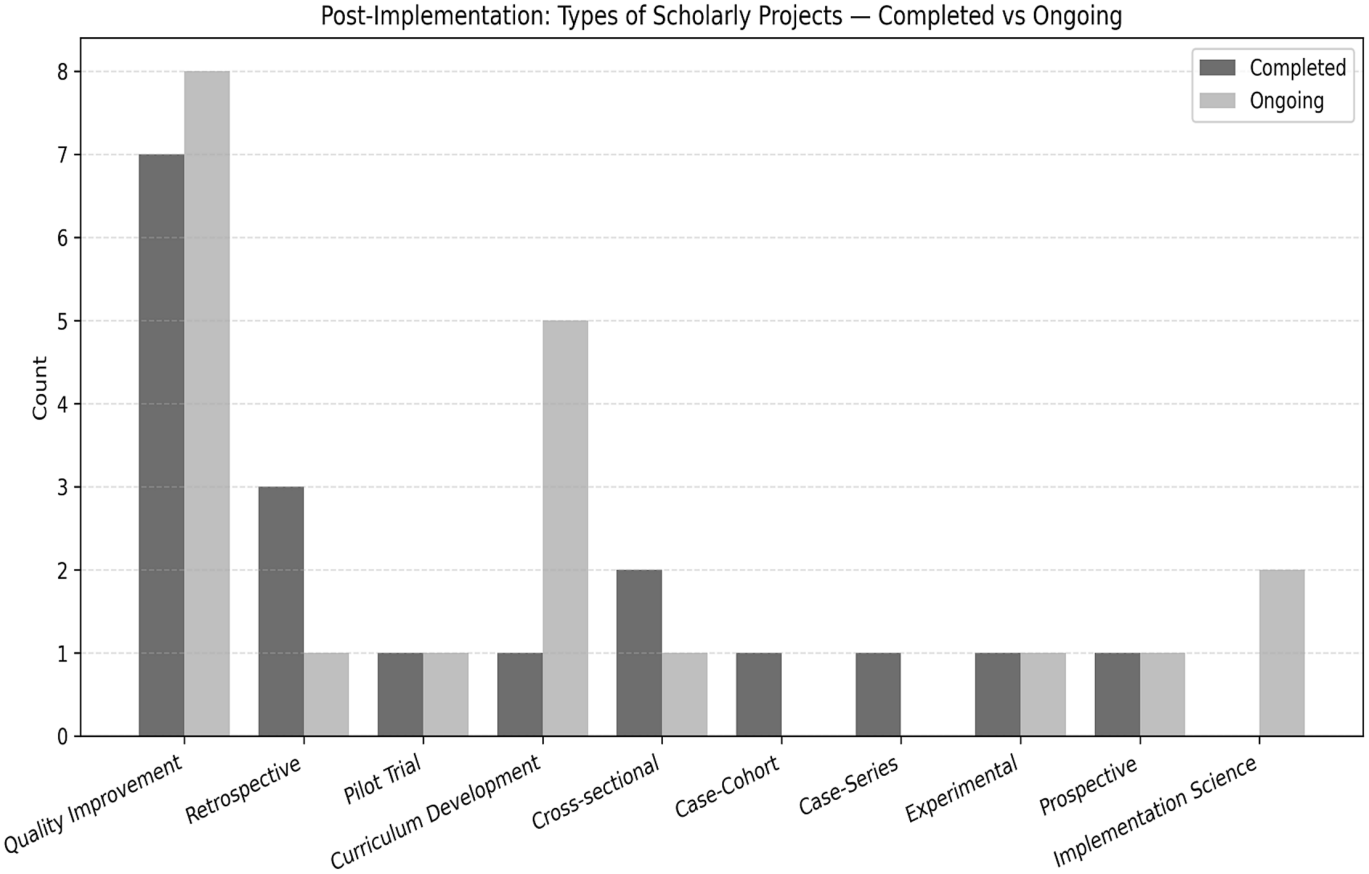
Eighteen projects were completed, while 20 initiatives remained ongoing at the time of analysis. Ongoing projects were at various stages of development, including data collection, IRB submission, protocol drafting, and data analysis. Quality improvement and curriculum development projects were the leading initiatives needing the most support from the faculty scholarship program.

Across all non‐physician scientist groups, including faculty, fellows, residents, attendings, and APPs, scholarly output demonstrated a marked rise following implementation regardless of program participation (Figure 3). Pre‐implementation scholarly activity among all groups totaled *N* = 85, while post implementation output increased to *N* = 311. Faculty demonstrated the most substantial increase, rising from *N* = 53 pre implementation to *N* = 248 post implementation. Growth was also observed among trainees and APPs: fellows increased output from *N* = 11 to *N* = 17, residents from *N* = 10 to *N* = 24, and APPs from *N* = 0 to *N* = 4. Attendings demonstrated increases as well, moving from *N* = 11 to *N* = 18 outputs post implementation. When comparing these trends to overall departmental productivity, the influence of physician scientists became clearer. Excluding the physician scientists in our analysis, pre‐implementation scholarly output was 26% lower than departmental totals that included them, *N* = 107. However, after implementation, the gap widened: scholarly output excluding physician scientists was 44% higher than overall total departmental output, *N* = 448. When referring to analyses that include vs. exclude physician‐scientists, our goal was to highlight that excluding this subgroup, an increase in faculty scholarship was displayed.

This analysis suggests how substantial post‐implementation gains among clinician educators were not solely attributable to physician scientist activity. Rather, these findings suggest the program provided targeted, meaningful support which translated into academic gains among clinician educators, those with historically fewer institutional resources and more limited protected time.

During the post‐implementation period, a total of 38 scholarly projects were actively supported through the faculty scholarship program. An additional indirect benefit of the program involved strengthening connections between our clinician educators and Vanderbilt's Community Engagement Studio (CES). As faculty sought support for patients facing community‐informed projects, the scholarship team facilitated referrals to CES to help investigators identify and engage appropriate patient advisors, stakeholders, or community members. This linkage enabled several clinician educators to refine research questions, enhance cultural and contextual relevance of study designs, and accelerate patient recruitment planning.

## 4 Discussion

In this department‐wide initiative, implementing a structured faculty scholarship program within an implementation science framework resulted in a meaningful increase in scholarly productivity among EM clinician educators. Program adoption of 17% of the target audience coincided with marked increases at both the department and participant levels, suggesting concentrated support, even when accessed by a subset of faculty and trainees.

Three components likely contributed to the observed gains. First, structured mentorship and accessible resources addressed well‐documented barriers for clinician educators, including limited time, unclear pathways for submissions to journals and conferences, and a lack of resources. Second, dedicated effort from two faculty physician scientists and a full‐time Health Services Research Analyst created visible infrastructure, a place to bring together ideas, obtain rapid feedback, and receive hands‐on assistance with drafting, formatting, and executing scholarly projects. Third, situating this initiative within the PRISM framework allowed iterative adjustments, which maintained high end‐user ratings. Together, these components likely lowered the burden of navigating scholarly work. In a recent study by Jordan et al. [18], a multivariate general linear model was used to examine differences in optimal time allocation across faculty development domains by site type, gender, and career stage. The mean optimal percentage of time for the domain of the faculty development domains of scholarship skills was 18% [18]. The three most frequently cited interests included collaborative research, writing for publication, and biostatistics [18]. These priorities were similarly identified in our needs assessment and addressed through structured mentorship, centralized methodological support, and guided dissemination resources. High acceptability within program participants indicates the program resonated with participants. By explicitly supporting non‐tenure track clinician educators who frequently face heavier clinical demands, the program may help reduce inequities in scholarship access and broaden pathways to promotion. Gains in abstracts and presentations can accelerate academic formation for clinician educators, gaining momentum for sustained scholarly engagement. Although faculty retention was not directly measured in this evaluation, fostering a supportive academic environment for clinician educators may improve faculty satisfaction and long‐term retention. In turn, improved retention has the potential to positively influence continuity, educational quality, and clinical outcomes within academic emergency departments.

### 4.1 Limitations

Despite our positive outcomes, there were several limitations. First, engagement in the program was voluntary, which may have introduced self‐selection bias. Faculty who engaged with the program may have been more motivated, more academically inclined, or already working on scholarly projects compared to non‐participants. As a result, the observed increases in adoptee output may not be fully generalizable to all clinician educators within the department. Second, the program's first‐year evaluation may have been limited by variable faculty availability and workload constraints. Competing clinical responsibilities and teaching commitments may have significantly limited the time faculty had to participate in scholarly activities, which in turn contributed to the modest adoption rate (17%). Third, all scholarly output data were extracted and verified by a single reviewer, which introduces the possibility of classification inconsistencies or data entry errors despite systematic cross‐references with multiple sources. Although this approach ensured uniform coding, the absence of duplicate review may reduce the reliability of the final counts. Fourth, incomplete or outdated ORCID profiles among 28 faculty members limited the accuracy of automated database verification. Since ORCID contains only peer‐reviewed journal publications, it did not capture all extensive scholarly activities commonly produced by EM clinician educators, including abstracts, educational materials, or online academic content. This exclusion may have resulted in undercounting or required greater reliance on CVs and PubMed searches, which vary in completeness. Fifth, while we predict that a similar increase in scholarly output would occur over the same time period, we cannot determine with certainty how much of the observed increase would have occurred with support from our program. We also acknowledge that while we expect our program to be largely responsible for an increase in scholarly output, we cannot be certain it was just time that passed. At last, the analysis focused primarily on quantitative productivity metrics without assessing the quality or academic impact of outputs. Measures such as journal impact factors, citation trajectories, or authorship equity were not included in our analysis. Future work will examine both the quality and impact of scholarly products to better understand the long‐term effectiveness of the faculty scholarship program and its contribution to academic promotion.

## 5 Conclusion

The implementation of a structured, resource‐integrated faculty scholarship program within a large academic ED resulted in a substantial increase in scholarly productivity and high levels of end‐user satisfaction. By directly addressing long‐standing barriers—such as limited mentorship, unclear processes, and fragmented institutional resources, the program created an accessible and sustainable pathway for clinician educators to develop, execute, and disseminate scholarly work. The 35.8% increase in departmental scholarly output, combined with improved productivity at the individual participant level, underscores the value of intentional, faculty scholarship efforts grounded in implementation science. As a result, clinician educators, who receive fewer dedicated resources than physician scientists, were able to build academic momentum and more efficiently navigate the complexities of scholarly production.

## Author Contributions

**Amina Belghit:** conceptualization, writing – original draft, methodology, validation, writing – review and editing, formal analysis, resources, data curation, visualization, project administration, investigation. **Jeremy S. Boyd:** conceptualization, methodology, writing – review and editing, project administration, resources. **Alan B. Storrow:** writing – review and editing, writing – original draft, methodology, visualization, formal analysis, conceptualization, supervision. **Deja M. Curry:** data curation, writing – review and editing, project administration, conceptualization, resources. **William B. Stubblefield:** writing – original draft, writing – review and editing, conceptualization, methodology, formal analysis, visualization, project administration. **Jim Olechna:** data curation, project administration, writing – review and editing, resources. **Erik P. Hess:** conceptualization, writing – review and editing, methodology, project administration.

## Funding

The authors have nothing to report.

## Disclosure

The authors have nothing to report.

## Conflicts of Interest

The authors declare no conflicts of interest.

## Data Availability

The data that support the findings of this study are available from the corresponding author upon reasonable request.
